# Scientific Evidence for Korean Medicine and Its Integrative Medical Research 2017

**DOI:** 10.1155/2018/1498020

**Published:** 2018-01-10

**Authors:** Wansu Park, Vesna Sendula-Jengic, Seong Su Nah, Han Chae

**Affiliations:** ^1^Department of Pathology, College of Korean Medicine, Gachon University, Seongnam, Republic of Korea; ^2^Psychiatric Hospital Rab, Rijeka University, Rab, Croatia; ^3^School of Medicine, Boston University, Boston, MA, USA; ^4^Division of Longevity and Biofunctional Medicine, School of Korean Medicine, Pusan National University, Busan, Republic of Korea

In 2016, the total medical fee for Korea Medicine (KM) in South Korea was over 4 billion dollars and the National Health Insurance of South Korea paid about 2.09 billion dollars for medical services provided by 19,737 KM doctors in 302 KM hospitals and 14,150 KM local clinics. Actually, 12.9 million Korean patients received 105 million outpatient services provided by KM doctors in 2016. The older the Korean society is, the more important the role of KM becomes, because Korea is anticipated to be a hyperaged society in 2028. As well as the* Donguibogam*, which was enlisted on the Memory of the Word by UNESCO in 2009, Sasang typology, Sasang Constitutional Medicine, Sa-am acupuncture, Chuna therapy, Pharmacopuncture, Korean physical therapy, Korean psychotherapy, and so on characterize KM. But more integrative researches and scientific evidences for KM might strengthen both clinical efficacy and applicability of KM. In this respect, it seems to be unavoidable that KM utilizes cutting-edge techniques of modern science. The more accurate and efficient practice of KM innovated with the modern bioscientific technology would help people live healthy lives.

Our special issue, which had opened for 6 months in the first half of 2017, focused on scientific evidence for KM and its integrative medical research.

An article by W.-W. Choi et al. described that the traumatic brain injury (TBI) mouse model was induced using the controlled cortical impact method; Chunghyul-Dan (CHD) was orally administered twice a day for 5 d after TBI induction; mice were assessed for brain damage, brain edema, blood-brain barrier (BBB) damage, motor deficits, and cognitive impairment; treatment with CHD reduced brain damage seen on histological examination and improved motor and cognitive functions; however, CHD did not reduce brain edema and BBB damage; CHD could be a candidate agent in the treatment of patients with TBI.

S. M. Hong et al. described that the aim of this study was to determine the differential effect of sleep deprivation in individuals with different body compositions (fluid) according to Soyang type (SY) and Taeeum type (TE); total body water and extracellular water were significantly different between the groups in the intervention phase; physiological parameters also varied from the beginning of the resting phase to the end of the experiment; potassium levels changed more in SY than TE individuals; participants responded differently to the same amount of sleep deprivation depending on their Sasang constitution types; this study indicated that SY individuals were more sensitive to sleep deprivation and were slower to recover from the effects of sleep deprivation than TE individuals.

J. Kim et al. described that they assessed the quality of reporting based on CAse REport (CARE) and STandards for Reporting Interventions in Clinical Trials of Acupuncture (STRICTA) guideline checklists; a total of 93 eligible case reports of acupuncture treatment were identified among the 107 articles screened; overall quality of reporting in the case reports was generally acceptable (75.4% on CARE, 67.7% on STRICTA), but several crucial items remained substantially underreported; in conclusion, endorsement of the CARE and STRICTA guidelines is needed to improve the completeness of reporting.

S.-R. Kim et al. described that they screened 189 participants aged 20 to 49 years, complaining of headache; to classify patients in terms of Yin deficiency, they used two self-reporting Yin-deficiency questionnaires and diagnosis by a doctor; based on the tests, a total of 33 subjects were assigned to a Yin-deficient group and 33 subjects were assigned to a nondeficient control group; tongue images were acquired using a computerized tongue diagnostic system, for evaluating tongue indices; in conclusion, Yin-deficient patients had less tongue coating and tended to have a more reddish tongue than nondeficient patients.

An interesting study by S. Jang et al. described that this survey aimed to investigate the characteristics of users and nonusers of herbal medicine and the adverse events experienced due to herbal medicines in South Korea; the questionnaire consisted of safety, using experience, using type, usage and nonusage reason, purchase location, and adverse events of herbal medicine; of the total 1,134 respondents, 726 (64.0%) considered herbal medicine safe, and 693 (61.1%) answered that they have taken herbal medicines within the past year; among those who took herbal medicines, 46 experienced adverse events, and the most frequently reported symptoms were digestive disorders (52.2%); regulation of herbal medicines is needed in order to resolve problems related to the safety of herbal medicines.

S. J. Jung et al. described that the distribution of mast cells (MCs) in the ventral skin of mice was studied so that it could be used to infer the locations, depths from the epidermis, and sizes of three putative acupuncture points (Aps); the harvested skins from 8-week-old mice were stained with toluidine blue, and the MCs were recognized by their red-purple stains and their metachromatic granules; the three putative APs, CV 8 and the left and the right KI 16 APs, were identified based on their high densities of MCs; these findings also imply that acupuncture may stimulate, through MCs, an immune response to allergic inflammation.

Y.-C. Yun et al. described that the Morris water maze (MWM) test demonstrated a significant improvement in hippocampal-dependent memory in the middle cerebral artery occlusion (MCAO) rats after laser acupuncture (LA) treatment; LA treatment significantly reversed the postischemic decrease in choline acetyltransferase immunoreactivity in the hippocampal CA1 region; LA treatment could improve cognitive impairment in MCAO rats to enhance the cholinergic system in the hippocampal CA1 region and to exert a neuroprotective effect by regulating Creb, Bdnf, Bcl-2, and Bax gene expressions.

J. Kim et al. described that their study aims to investigate the efficacy of Yukgunja-tang (YGJT) on functional dyspepsia (FD) patients classified by 3-dimensional facial measurement using a 3-dimensional facial shape diagnostic system (3-FSDS); a placebo-controlled, double-blind, randomized, two-center trial will be performed to evaluate the efficacy of YGJT on FD patients; the results of this trial will help the FD patients improve the symptoms and quality of life effectively and provide objective evidence for prescribing the YGJT to FD patients in clinical practice; this trial is registered with Clinical Research Information Service Identifier: KCT0001920.

S. Mun et al. described that data on cold (CP), heat (HP), spleen-qi deficiency (SQDP), and kidney deficiency (KDP) patterns were extracted by a factor analysis of symptoms experienced by 954 participants; the CP and SQDP scores were higher and the HP score was lower in women; the HP and SQDP scores decreased with age, while KDP scores increased with age; the underlying pathology of CP and SQDP might be associated with the body's metabolic rate.

In conclusion, we expect that this special issue updates scientific evidences in KM integrative research and makes a useful progress for improving KM practice.

